# Supplementary material for: Training a force field for proteins and small molecules from scratch
Source: Chem Sci. 2026 Sep 17;17(42):20573–94. doi: 10.1039/d6sc02874h (PMC13585093; doi:10.1039/d6sc02874h)
Supplement: Supplementary file 1 [file SC-017-D6SC02874H-s002.pdf]

Supplementary Information (SI) for Chemical Science.
This journal is © The Royal Society of Chemistry 2026

Training a force field for proteins and small molecules from scratch

Supplementary Data

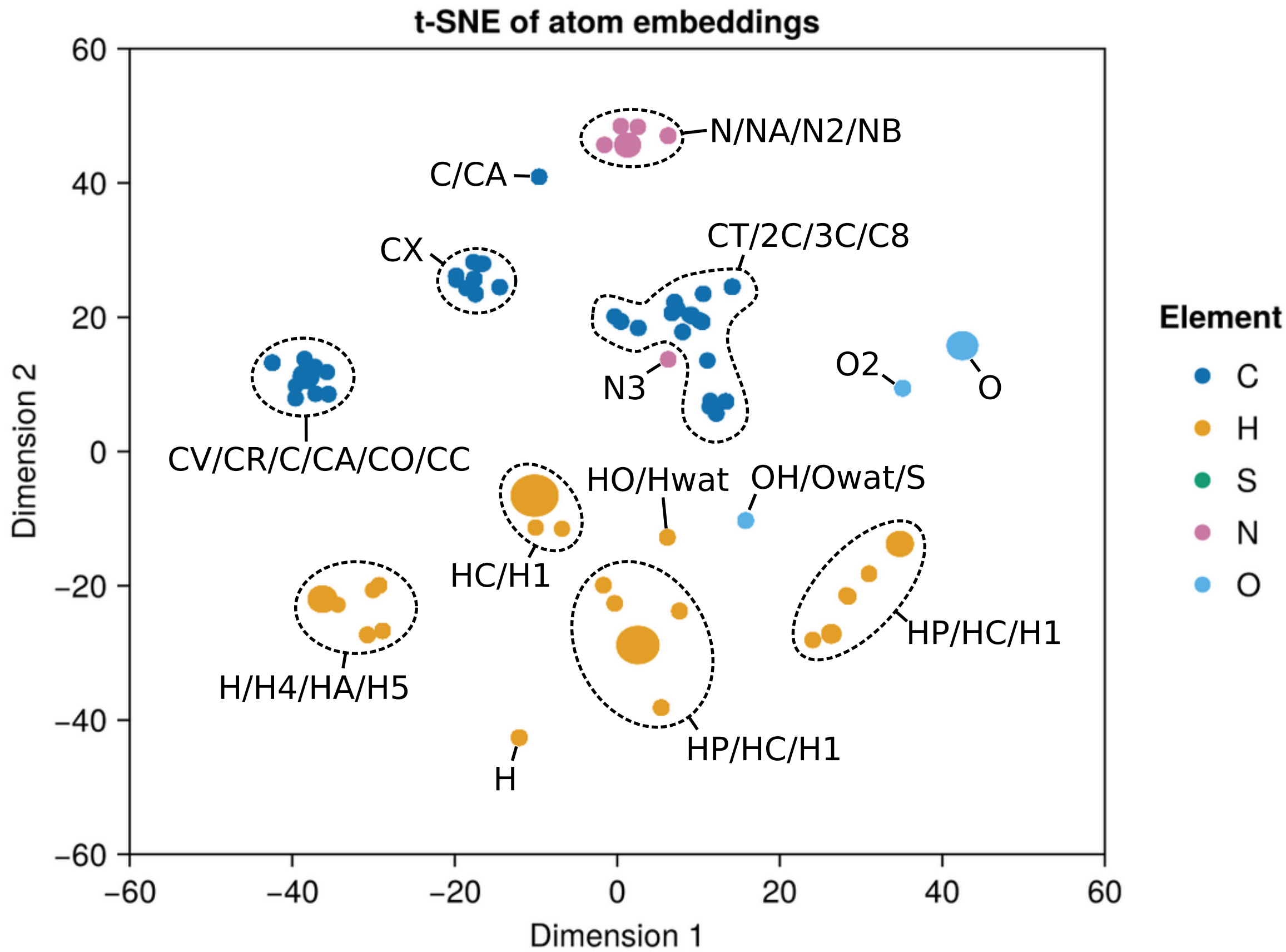

Supplementary Figure 1 Exploring the embedding space of continuous atom types. Ubiquitin was parameterised with Garnet and the 64-dimensional embedding of each atom was calculated. t-SNE was used to reduce this to two dimensions for visualisation. The corresponding Amber14SB atom types found in different regions are shown, along with water atoms (Owat and Hwat).

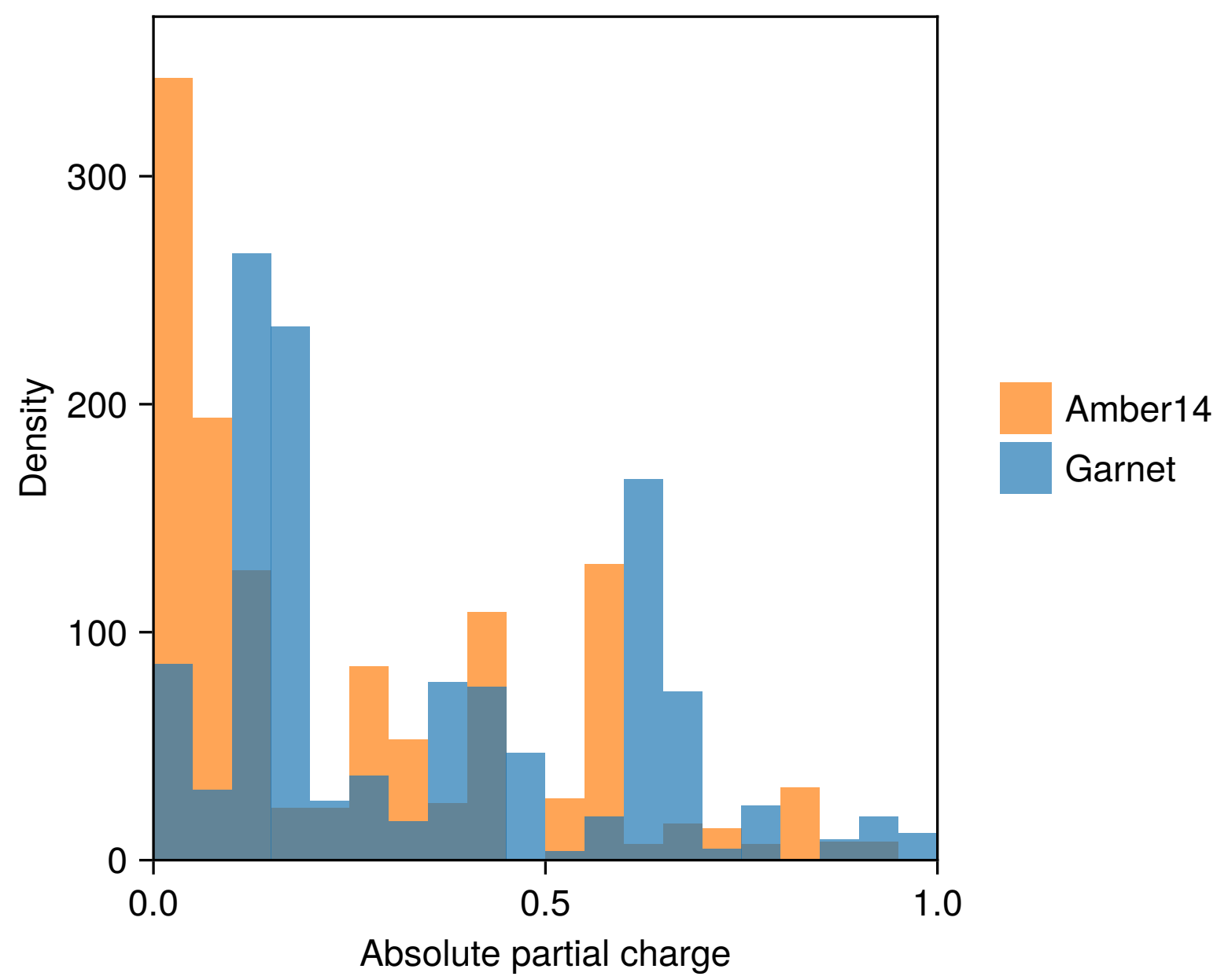

Supplementary Figure 2 Partial charge distribution in Garnet. The partial charge of each atom in ubiquitin (1,231 atoms) is assigned with Garnet or Amber14SB [2]; the distribution of the absolute values of the partial charges is shown. For Garnet, the mean is 0.33 and the median is 0.19. For Amber14SB, the mean is 0.25 and the median is 0.11.

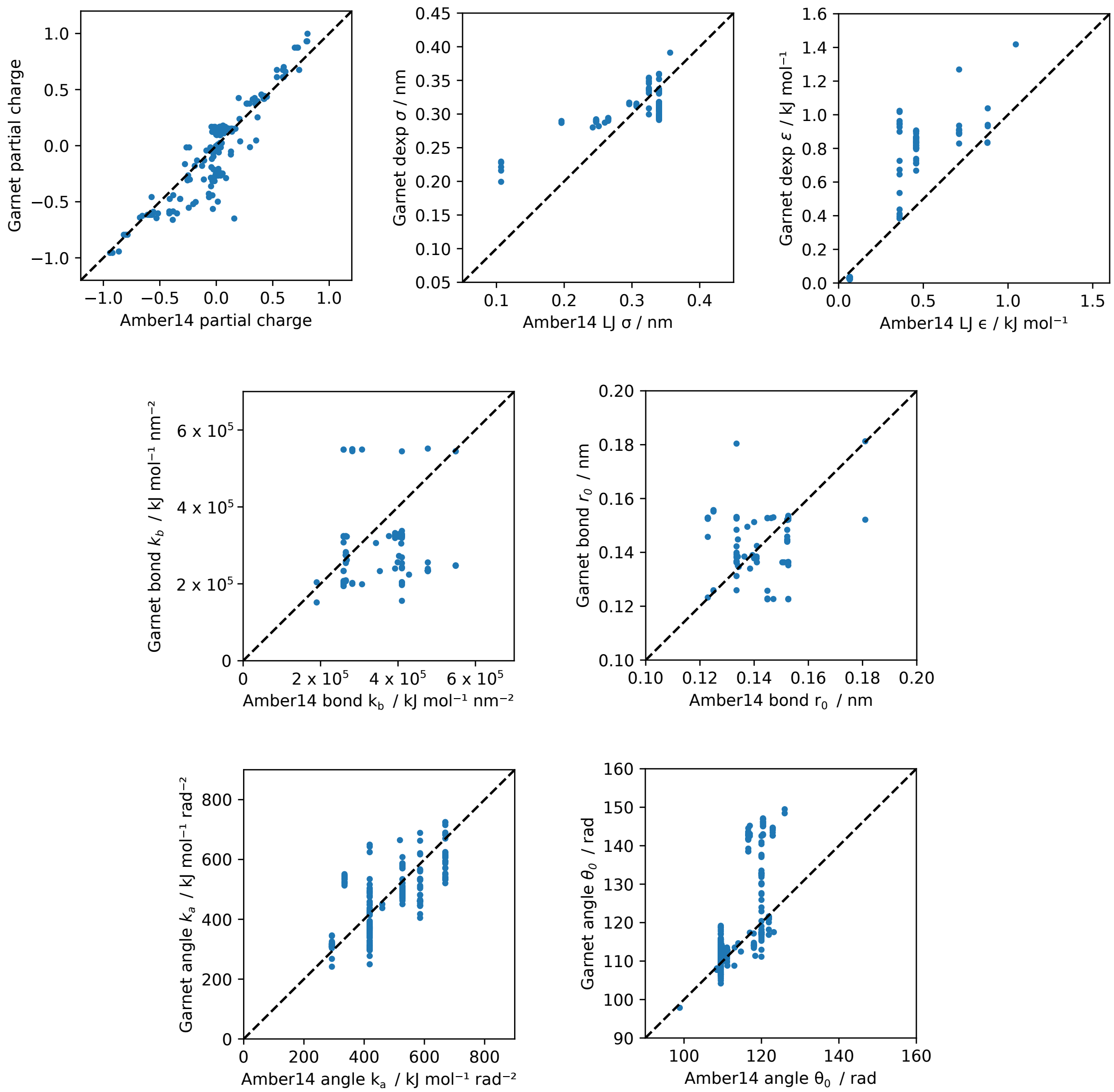

Supplementary Figure 3 Comparison of parameters assigned to ubiquitin by Garnet and Amber14SB [2]. The functional forms of the Lennard-Jones and double exponential potentials are different, with σ and ε playing a similar role in both.

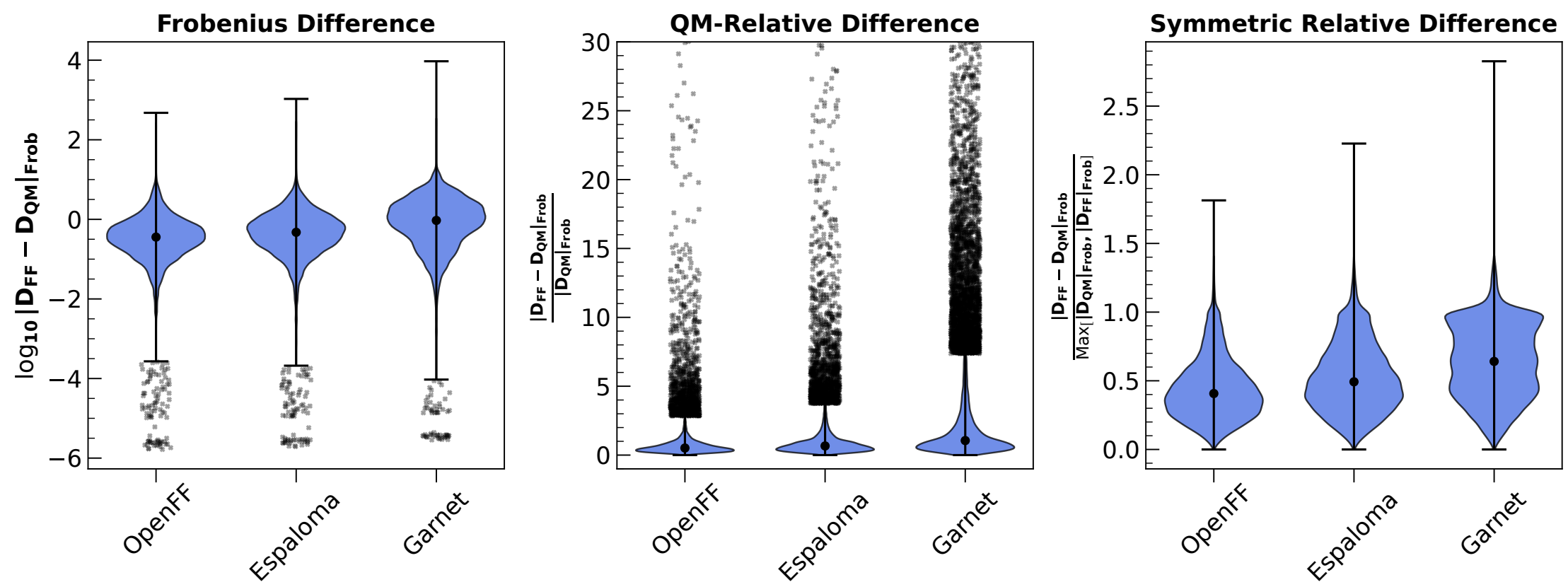

Supplementary Figure 4 Comparison of dipole moments between OpenFF, Espaloma and Garnet, expressed as Frobenius norm differences of the dipole tensor.

Motif	N_{all}	OpenFF	Espaloma	MACE-OFF	Garnet
pyrazole	11163	–	1.41 [1.22, 1.62]	2.41 [1.44, 3.71]	1.27 [1.10, 1.48]
aldehyde	78	8.24 [3.69, 18.53]	16.24 [8.72, 30.43]	–	6.78 [2.87, 16.30]
thiol	37	5.65 [1.63, 21.25]	5.65 [1.63, 21.23]	–	5.65 [1.63, 21.24]
pyrrole	7533	–	1.29 [1.07, 1.55]	–	1.28 [1.05, 1.54]
phenol	6044	2.47 [2.13, 2.86]	–	–	1.33 [1.08, 1.63]
carboxylic_acid	3160	–	1.49 [1.13, 1.96]	–	1.62 [1.24, 2.11]
indole	3068	–	1.71 [1.31, 2.21]	–	1.74 [1.34, 2.26]
alkyne	1339	1.88 [1.27, 2.77]	14.48 [12.52, 16.69]	–	–
diazo	240	2.97 [1.43, 6.17]	–	32.33 [11.15, 88.06]	–
fused_aromatic	10538	1.79 [1.57, 2.02]	–	–	–
imidazole	10014	–	–	–	1.47 [1.27, 1.70]
fluorinated_C	8871	–	–	–	1.31 [1.10, 1.55]
ketone	3266	–	–	–	1.95 [1.53, 2.45]
indazole	2144	–	1.59 [1.14, 2.21]	–	–
carboxylate	1601	1.82 [1.27, 2.61]	–	–	–
oxazole	1346	–	2.56 [1.84, 3.56]	–	–
imide	969	–	–	–	2.19 [1.43, 3.32]
azide	16	32.96 [11.78, 98.23]	–	–	–

Supplementary Table 1 Small molecule groups that appear more in the lowest 1% of molecules by structure RMSD to QM structures after minimisation. The numbers represent the median and 95% confidence bounds of the enrichment.

Motif	N_{all}	OpenFF	Espaloma	MACE-OFF	Garnet
amide	37001	1.30 [1.22, 1.37]	1.41 [1.34, 1.48]	–	1.46 [1.39, 1.53]
tertiary_amine	21595	1.87 [1.75, 2.00]	1.34 [1.23, 1.47]	–	1.38 [1.26, 1.50]
sulfonamide	5463	–	1.45 [1.17, 1.78]	2.67 [1.59, 4.06]	1.58 [1.29, 1.93]
quaternary_amine	4899	1.42 [1.13, 1.77]	1.73 [1.41, 2.11]	–	1.64 [1.33, 2.02]
sulfone	9281	–	–	2.53 [1.59, 3.70]	1.29 [1.09, 1.51]
aniline	6218	1.45 [1.19, 1.76]	–	–	1.29 [1.05, 1.59]
ester	5876	1.69 [1.41, 2.03]	1.71 [1.42, 2.05]	–	–
disulfide	57	–	9.51 [3.99, 23.03]	–	9.51 [3.99, 23.02]
pyrimidine	16159	1.31 [1.16, 1.47]	–	–	–
ether	13971	–	1.41 [1.24, 1.58]	–	–
alkene	7265	–	–	–	1.87 [1.60, 2.18]
nitrile	4298	1.35 [1.05, 1.74]	–	–	–
urea	3229	–	–	–	1.94 [1.52, 2.46]
indazole	2144	–	2.27 [1.72, 2.98]	–	–
isoxazole	1472	–	1.71 [1.16, 2.52]	–	–
oxazole	1347	–	–	–	2.02 [1.39, 2.93]
conjugated	722	–	–	–	2.67 [1.70, 4.15]
sulfoxide	588	–	–	2.37 [1.48, 3.45]	–
guanidine	436	–	3.04 [1.76, 5.23]	–	–
enol	222	–	3.70 [1.90, 7.29]	–	–
phosphonate	82	–	–	–	6.43 [2.76, 15.42]
enoxide	34	–	6.18 [1.81, 23.03]	–	–

Supplementary Table 2 Small molecule groups that appear more in the highest 1% of molecules by structure RMSD to QM structures after minimisation. The numbers represent the median and 95% confidence bounds of the enrichment.

Property	Metric	Unit	GAFF	OpenFF-2.2.1	Garnet
ΔH_v	RMSE (Train)	kcal/mol	1.14	0.36	0.13
	RMSE (Test)	kcal/mol	1.67 [0.56, 2.72]	2.63 [0.48, 4.48]	4.29 [2.12, 6.12]
	Pearson's r (Test)	-	0.88 [0.74, 0.99]	0.84 [0.69, 0.99]	0.81 [0.61, 0.97]
ρ	RMSE (Train)	g/mL	0.045	0.017	0.045
	RMSE (Test)	g/mL	0.021 [0.010,0.030]	0.041 [0.021, 0.058]	0.13 [0.11, 0.16]
	Pearson's r (Test)	-	0.99 [0.99,0.99]	0.99 [0.98, 0.99]	0.93 [0.87, 0.99]

Supplementary Table 3 Performance of GAFF, OpenFF-2.2.1 and Garnet on predicting small molecule properties ΔH_v and ρ. RMSE and Pearson's r between experimental and calculated ΔH_v and ρ data are shown for the three force fields and separately for Garnet test and training data (ΔH_v values for three molecules were seen during training). The force fields were evaluated on the three training molecules as well as on 12 independent test molecules. Two test molecules had to be excluded from the GAFF evaluation, see the Methods. Numbers in brackets indicate bootstrapped 95% confidence intervals. Since the training dataset consisted of only three molecules, bootstrapped confidence intervals and r values are not reported for the training data.

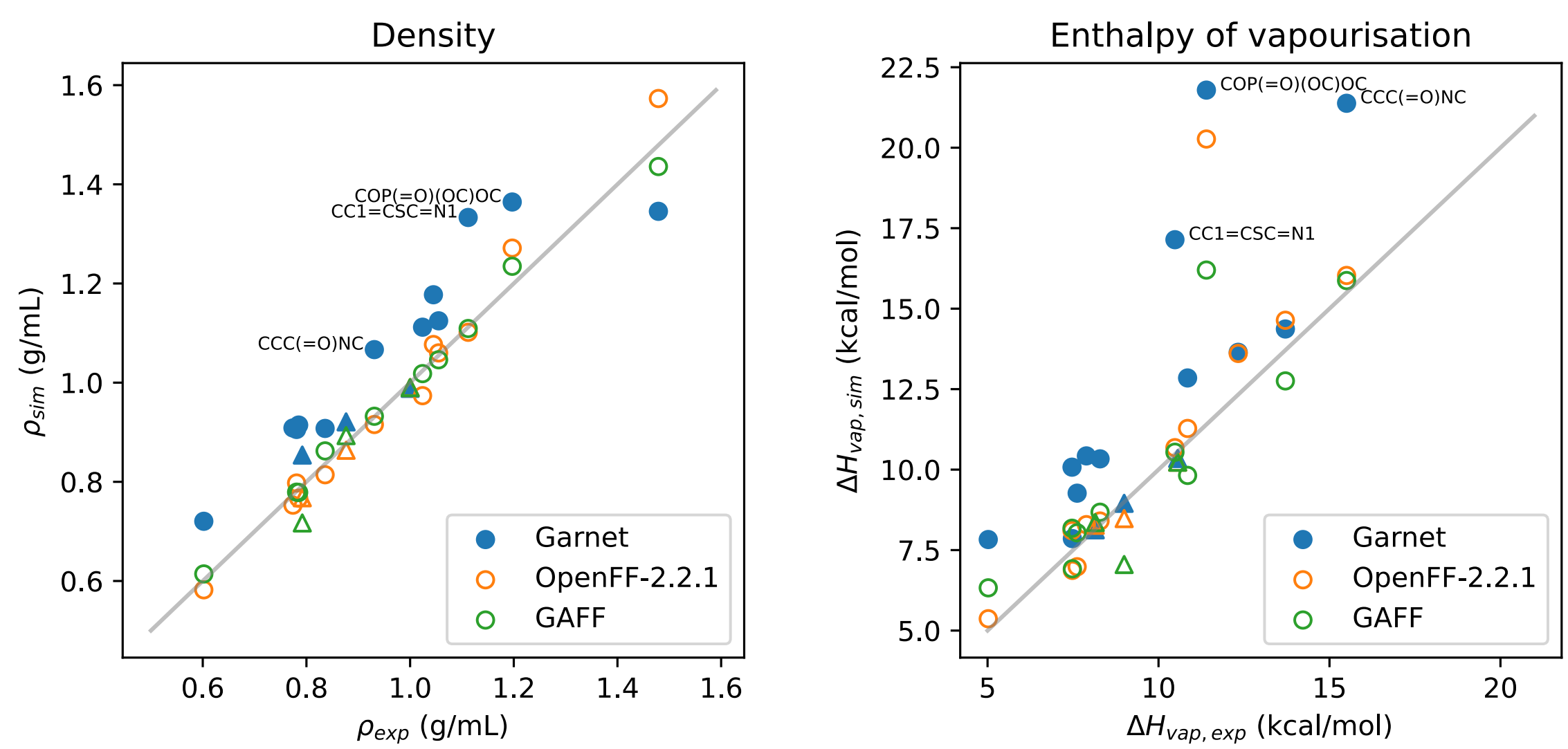

Supplementary Figure 5 Performance of GAFF (green), OpenFF-2.2.1 (orange) and Garnet (blue) on predicting density (ρ) and enthalpy of vapourisation (ΔH_v) for small molecules. Triangles indicate that ΔH_v data was used for Garnet training. SMILES strings are indicated for the three small molecules for which ΔH_v is considerably overestimated by Garnet.

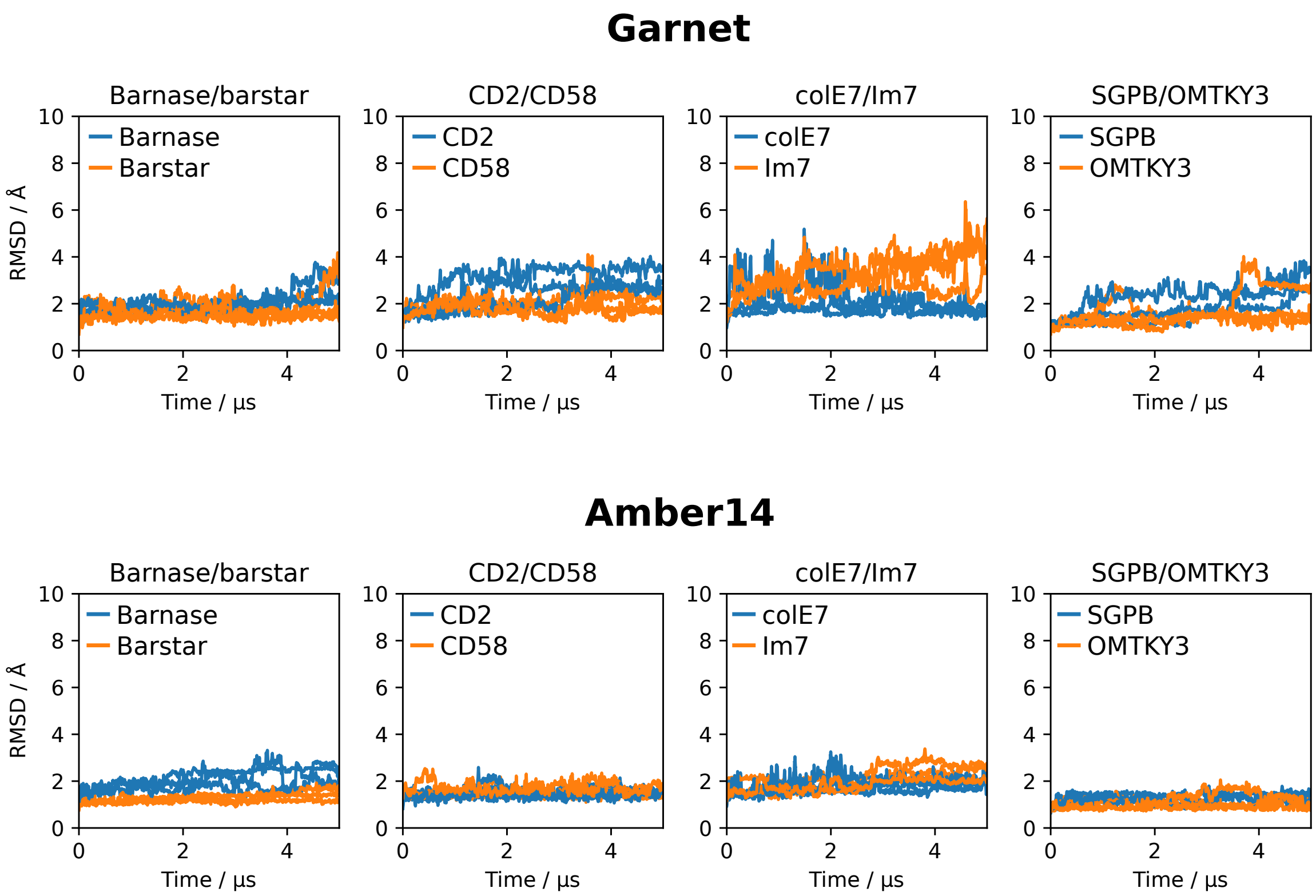

Supplementary Figure 6 Performance of Garnet on simulating protein complexes. The simulations are the same as in Figure 4A. The RMSD of each monomer to its starting conformation is shown over the course of three repeats. The RMSD is smoothed by taking the mean over a window of values extending 10 snapshots either side.

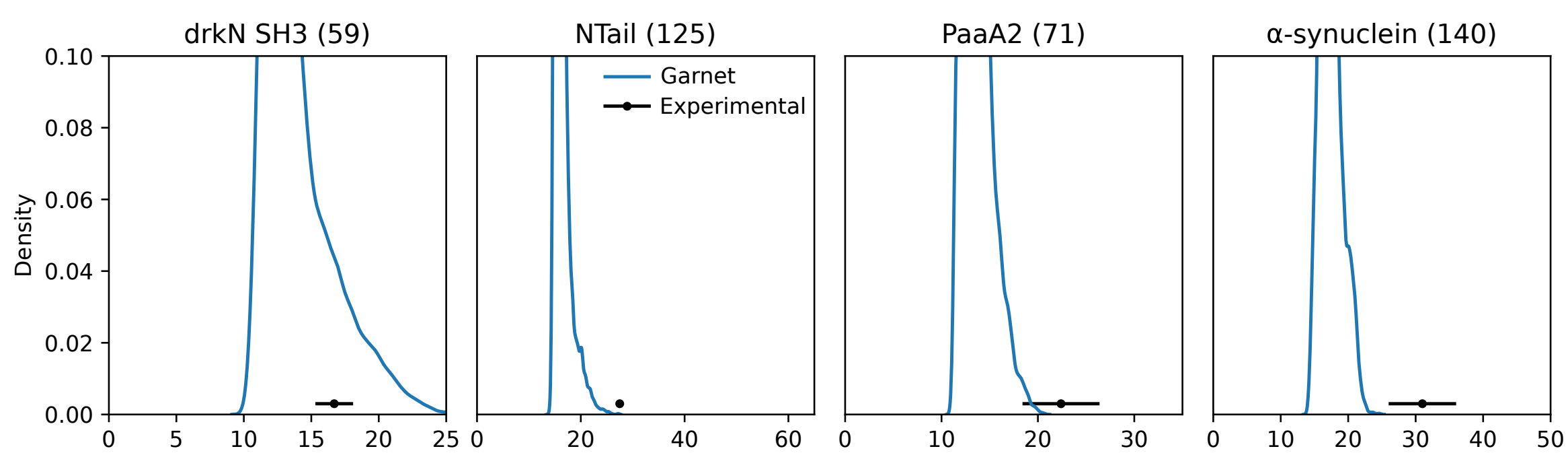

Supplementary Figure 7 Radius of gyration when simulating IDPs with Garnet. The simulations are the same as in Figure 4B-C. The R_g density is shown over the course of three repeats. The number in brackets is the number of residues in the protein.

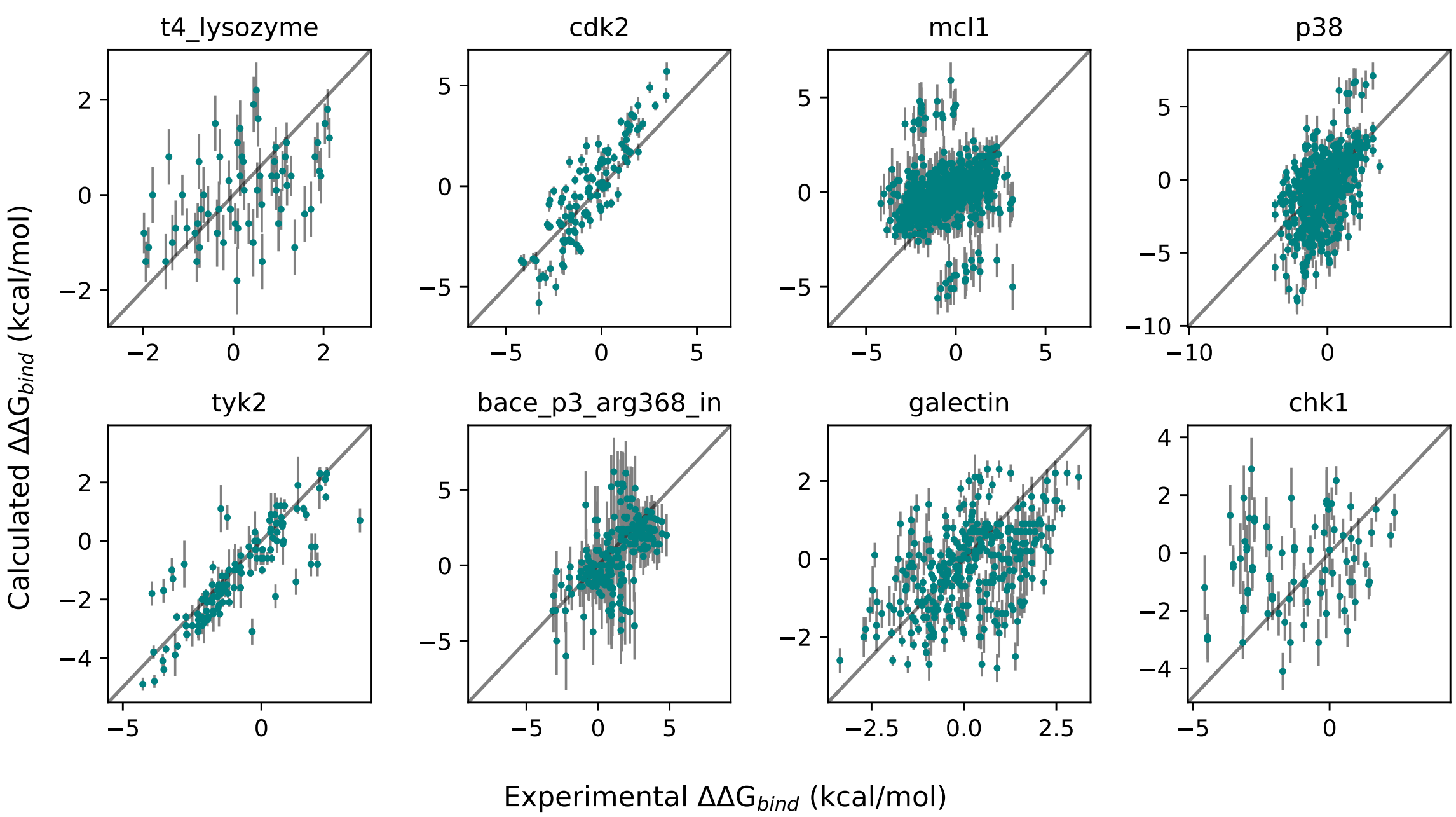

Supplementary Figure 8 Garnet RBFE performance. Calculated and experimental $\Delta\Delta G_{\text{bind}}$ values are shown for ligands binding to the eight proteins in our RBFE benchmark set. All (non-redundant) pairwise $\Delta\Delta G_{\text{bind}}$ values are shown per ligand series (i.e. per protein). These data were used to estimate the RMSE values reported for Garnet in Figure 6. Error bars are shown only for calculated $\Delta\Delta G_{\text{bind}}$ and report uncertainties as calculated by OpenFE.

Force field	Pairwise RMSE (kcal/mol)	ΔG Kendall τ	Fraction of best ligands
OpenFE	1.99 [1.82, 2.16]	0.63 [0.38, 0.82]	0.55
FEP+	1.07 [1.00, 1.14]	0.79 [0.66, 0.91]	0.74
Garnet	8.26 [7.74, 8.77]	-0.11 [-0.36, 0.17]	0.16

Supplementary Table 4 RBFE results for protein cmet. A single ligand transformation in the ligand network for cmet involved a net charge change of 1, the effect of which was not well predicted by Garnet. The poorly-predicted edge singly connected all charged to all uncharged ligands in the transformation network. A poor $\Delta\Delta G_{\mathrm{bind}}$ estimate for the net charge change transformation caused $\Delta\Delta G_{\mathrm{bind}}$ estimates across the network to show a relatively large deviation from the experimental data, even though $\Delta\Delta G_{\mathrm{bind}}$ values between ligands of similar charges were relatively well-predicted (Supplementary Figure 9). Numbers in square brackets indicate bootstrapped 95% confidence intervals.

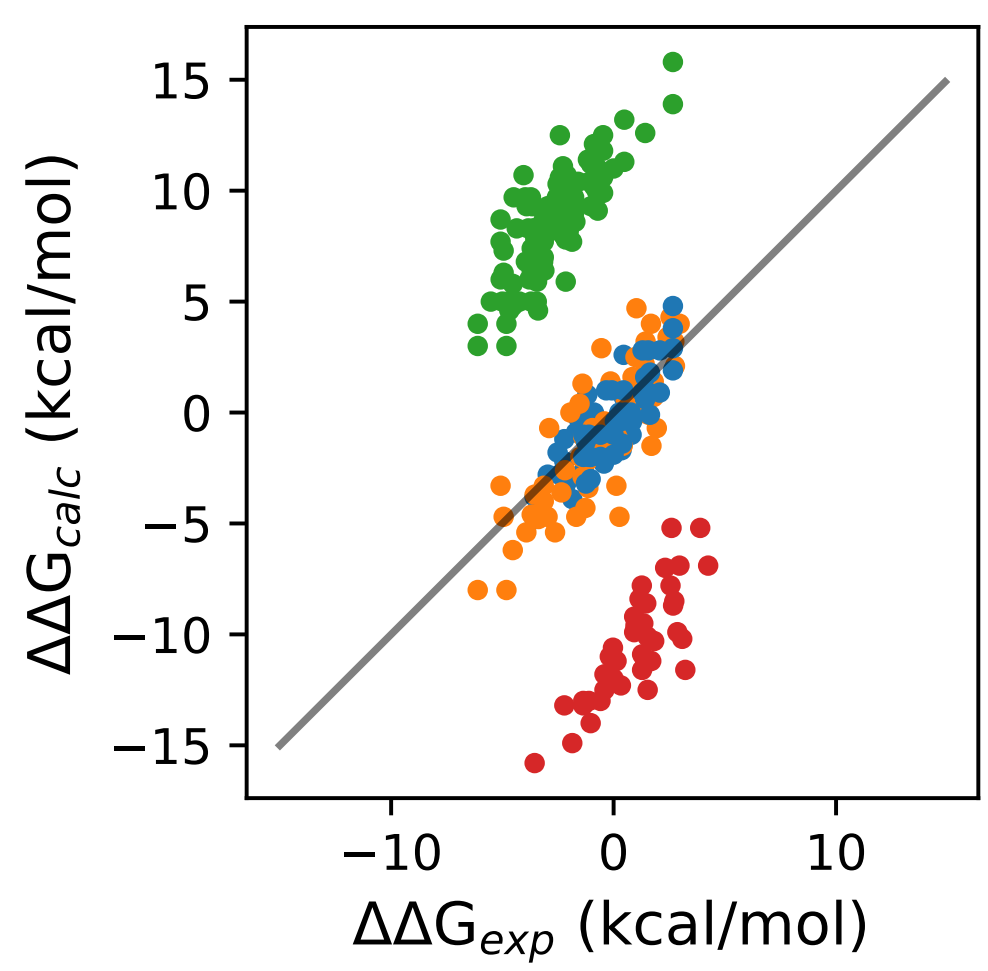

Supplementary Figure 9 Garnet RBFE performance for the protein cmet. Calculated and experimental $\Delta\Delta G_{\mathrm{bind}}$ values are shown. Half of the cmet ligands were neutral, and the other half of the ligands had a net charge of +1. ΔΔG values were relatively well-predicted (Kendall's τ = 0.63) for pairs of ligands with identical net charges (orange and blue data points). Absolute deviations to the experimental data were large for pairs with net charge differences (red and green data points), although correlations within each population of charge-changing pairs were relatively high (Kendall's τ = 0.60 for red cluster, Kendall's τ = 0.56 for green cluster). Ligand pairs are colour-coded using orange for neutral pairs, blue for pairs in which both ligands have a net charge of +1, green for pairs where the first ligand is neutral and the second charged, and red for pairs where the first ligand is charged and the second neutral.

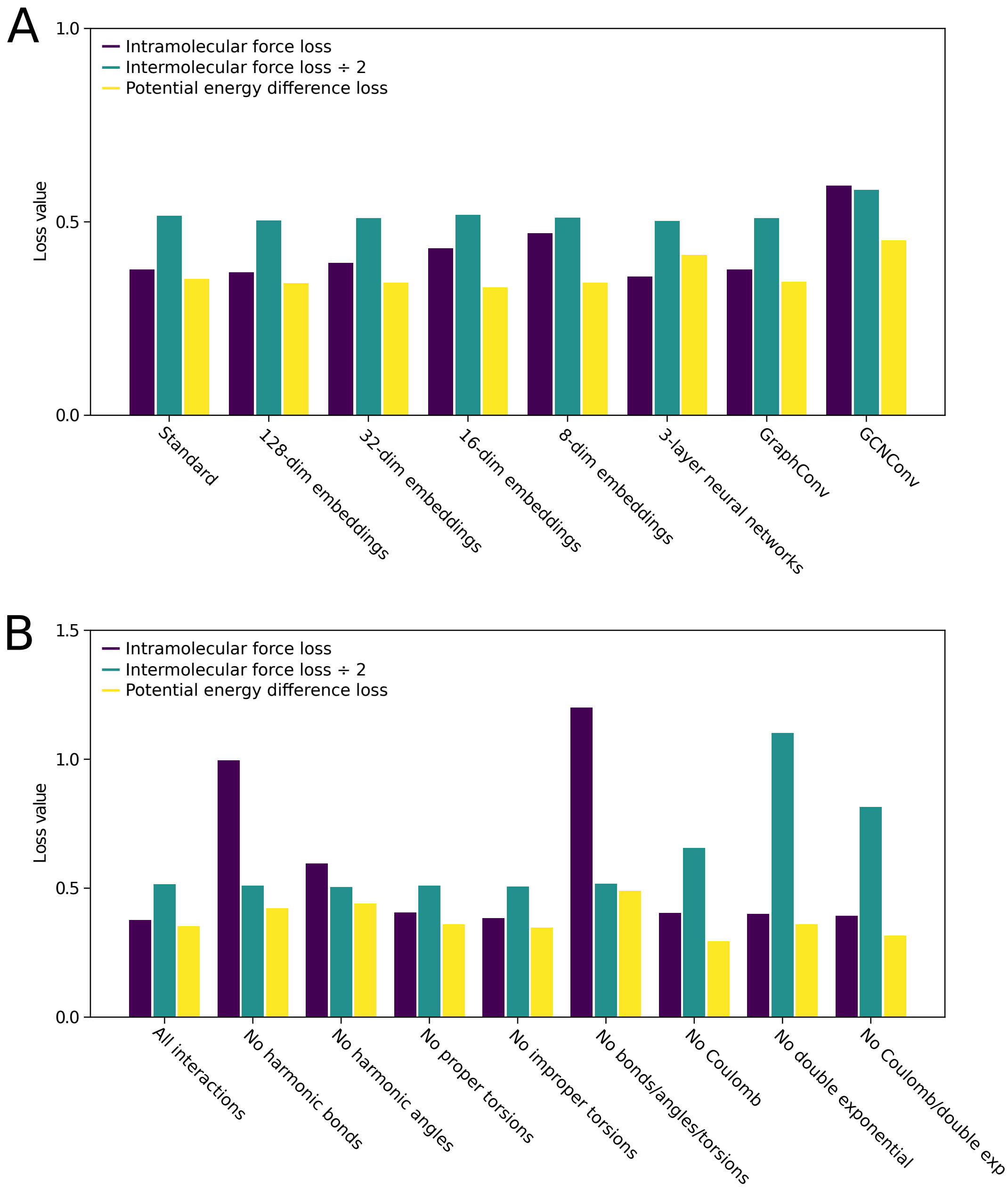

Supplementary Figure 10 Assessing the performance of model variants and training models without certain interactions. For each variant 4 models were trained from scratch with no training simulations run, i.e. only the reference trajectories were used. This is different to the full training runs in Figure 5C. The lowest total loss of the 4 models after 7 epochs of training was calculated to select the best model shown here. For ease of visualisation, the intermolecular force loss is scaled. (A) Neural network hyperparameters. The standard case used 64-dimensional embeddings, 2-layer neural networks and the GraphSAGE convolution layer [61]. (B) Removing components of the MM functional form. The double exponential potential is analogous to the Lennard-Jones potential and includes repulsive and attractive terms.

Dataset	n confs	Weighting	n confs used
SPICE Dipeptides Single Points Dataset v1.3	33,850	10	338,500
SPICE Solvated Amino Acids Single Points Dataset v1.1	1,300	100	130,000
SPICE DES370K Single Points Dataset v1.0 and v1.1	345,676	1	345,676
SPICE DES Monomers Single Points Dataset v1.1	18,700	1	18,700
SPICE PubChem Single Points Dataset v1.3	1,398,566	1	1,398,566
SPICE Solvated PubChem Set 1 v1.0	13,934	20	278,680
SPICE Amino Acid Ligand v1.0	194,174	2	388,348
SPICE Ion Pairs Single Points Dataset v1.2	1,426	10	14,260
SPICE Water Clusters v1.0	1,000	100	100,000
SPICE PubChem Boron Silicon v1.0	170,799	1	170,799
RNA Single Point Dataset v1.0	8,560	10	85,600
RNA Nucleoside Single Point Dataset v1.0	120	10	1,200
RNA Trinucleotide Single Point Dataset v1.0	6,080	10	60,800
MACE-OFF water	1,681	100	168,100
GEMS crambin	5,140	100	514,000
Total	2,201,006	-	4,013,229

Supplementary Table 5 DFT datasets used for training. The datasets are from SPICE [40, 41], Espaloma [16], MACE-OFF [42] and GEMS [73]. To ensure even training across all subsets, the data is weighted so that some subsets appear more often during training. Some of the data is retained as validation and test sets as described in the methods.

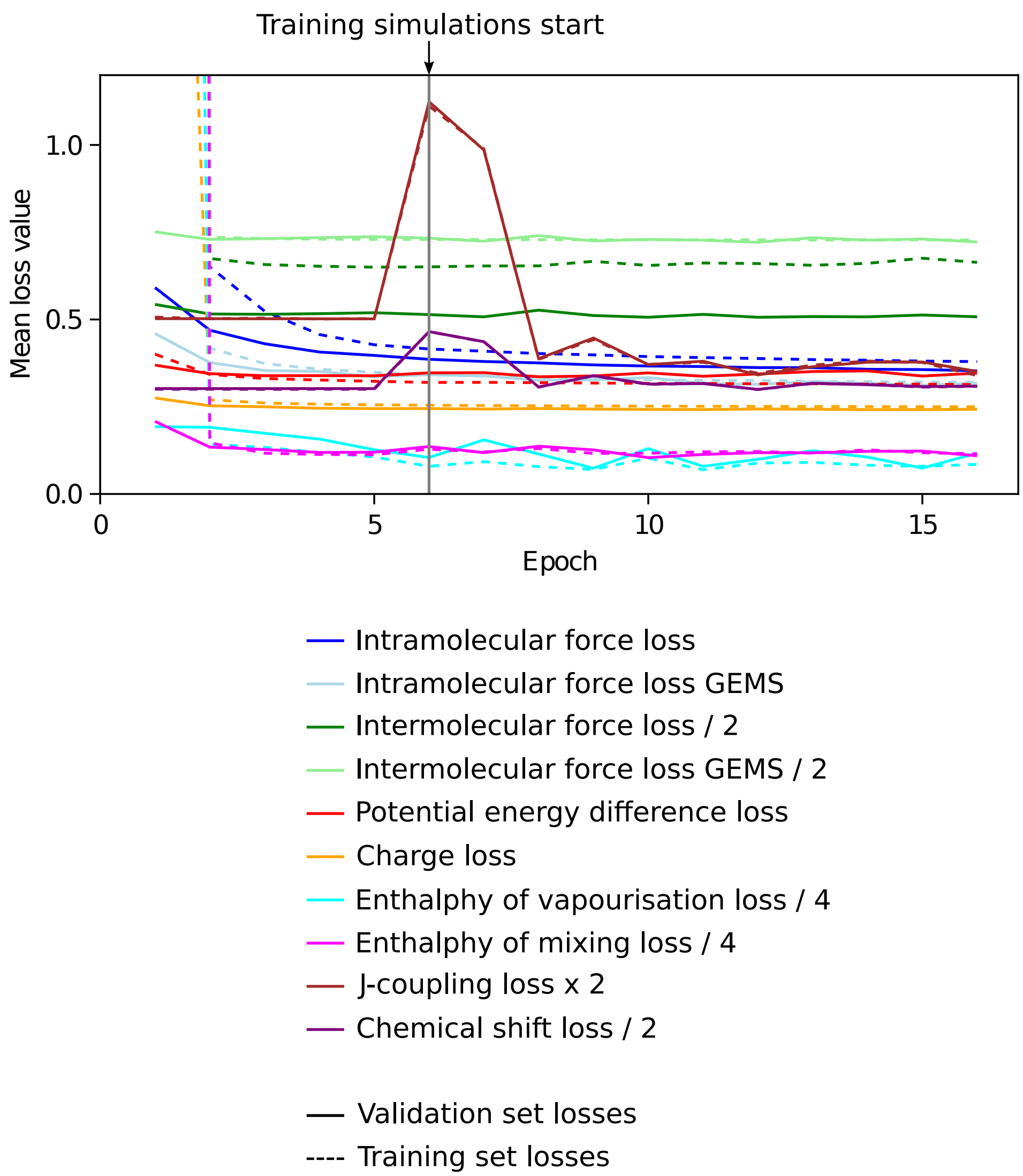

Supplementary Figure 11 Garnet training progress. The various loss values are plotted over the course of training. Solid lines correspond to the validation set and dotted lines to the training set. Some losses are scaled as noted for ease of visualisation. Reference trajectories are used to train on condensed phase and protein properties before epoch 6. From epoch 6, simulations run with the training force field are used. The model after epoch 12 was treated as the final model and used for benchmarking.
