# Supplementary material for: Training a force field for proteins and small molecules from scratch
Source: Chem Sci. 2026 Sep 17;17(42):20573–94. doi: 10.1039/d6sc02874h (PMC13585093; doi:10.1039/d6sc02874h)
Supplement: Supplementary file 2 [file SC-017-D6SC02874H-s001.zip › SI Figures/ablations.pdf]

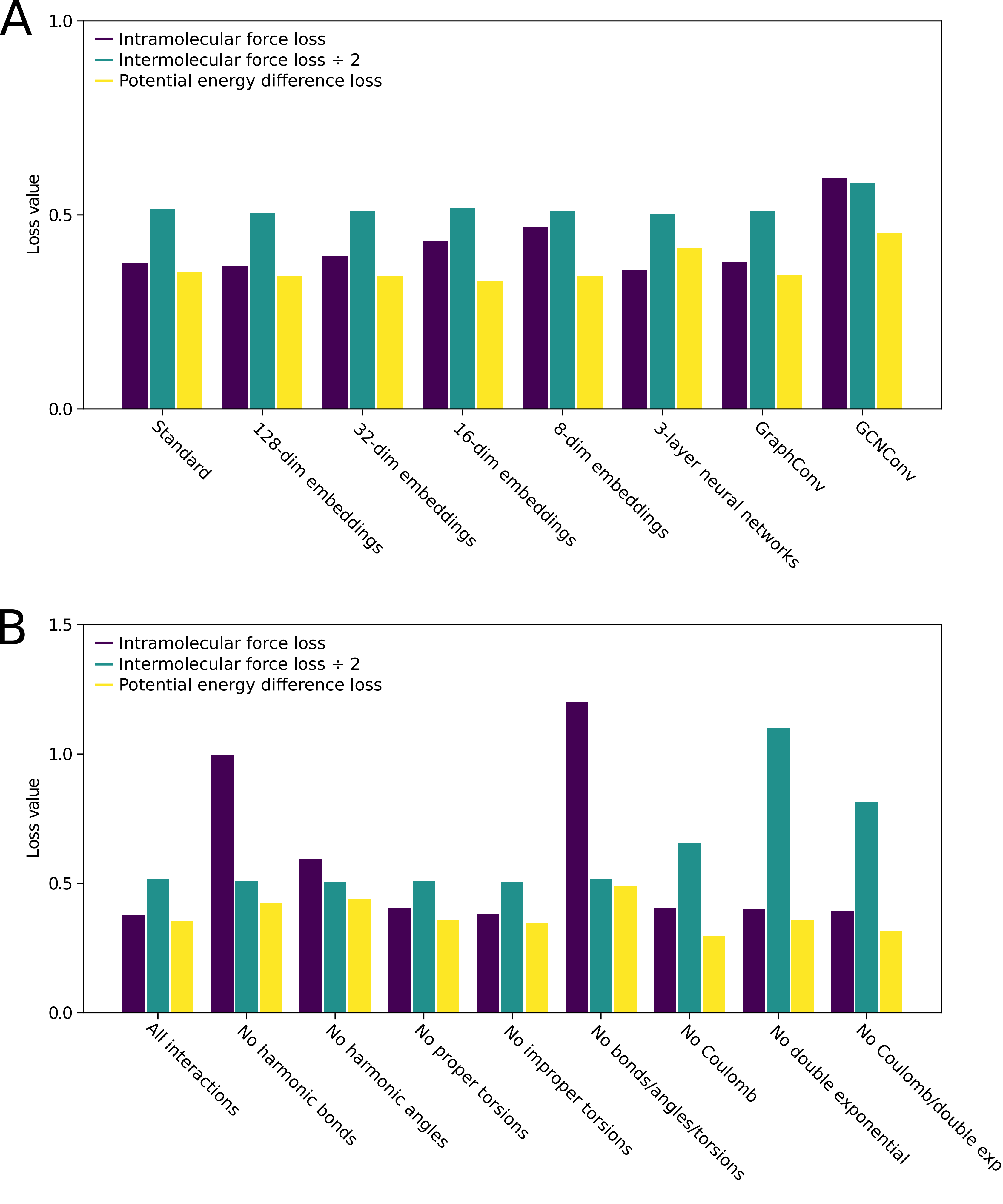
A
1.0
Intramolecular force loss
Intermolecular force loss ÷ 2
Potential energy difference loss
Loss value
0.5
0.0
Standard
128-dim embeddings
32-dim embeddings
16-dim embeddings
8-dim embeddings
3-layer neural networks
GraphConv
GCNConv
B
1.5
Intramolecular force loss
Intermolecular force loss ÷ 2
Potential energy difference loss
1.0
Loss value
0.5
0.0
All interactions
No harmonic bonds
No harmonic angles
No proper torsions
No improper torsions
No bonds/angles/torsions
No Coulomb
No double exponential
No Coulomb/double exp
