# Supplementary material for: Training a force field for proteins and small molecules from scratch
Source: Chem Sci. 2026 Sep 17;17(42):20573–94. doi: 10.1039/d6sc02874h (PMC13585093; doi:10.1039/d6sc02874h)
Supplement: Supplementary file 2 [file SC-017-D6SC02874H-s001.zip › SI Figures/complex_monomers.pdf]

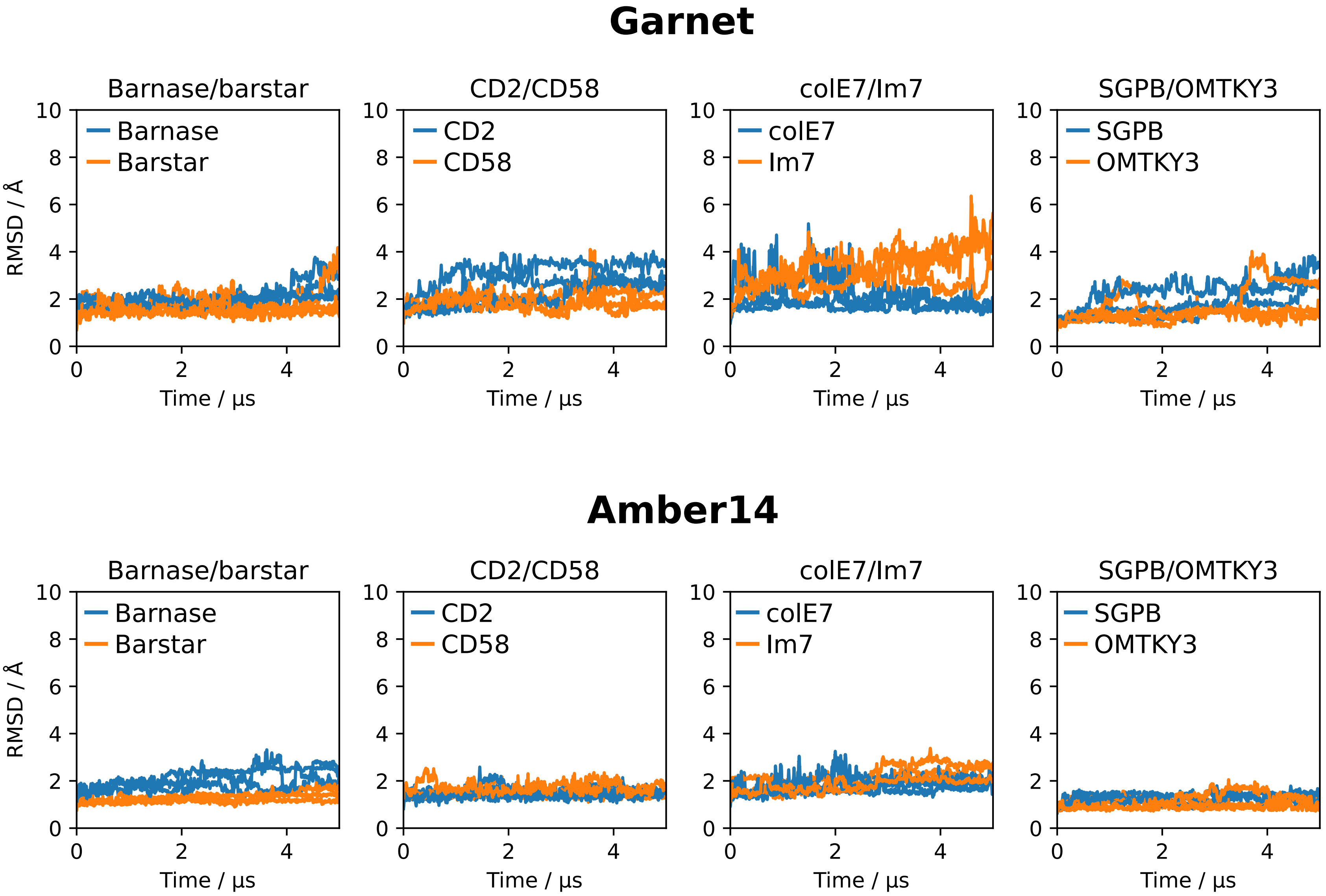
Garnet
Barnase/barstar
CD2/CD58
colE7/Im7
SGPB/OMTKY3
Barnase
Barstar
CD2
CD58
colE7
Im7
SGPB
OMTKY3
RMSD / Å
Time / μs
Amber14
Barnase/barstar
CD2/CD58
colE7/Im7
SGPB/OMTKY3
Barnase
Barstar
CD2
CD58
colE7
Im7
SGPB
OMTKY3
RMSD / Å
Time / μs
