# Supplementary material for: Training a force field for proteins and small molecules from scratch
Source: Chem Sci. 2026 Sep 17;17(42):20573–94. doi: 10.1039/d6sc02874h (PMC13585093; doi:10.1039/d6sc02874h)
Supplement: Supplementary file 2 [file SC-017-D6SC02874H-s001.zip › SI Figures/ddg_exp_calc_cmet.pdf]

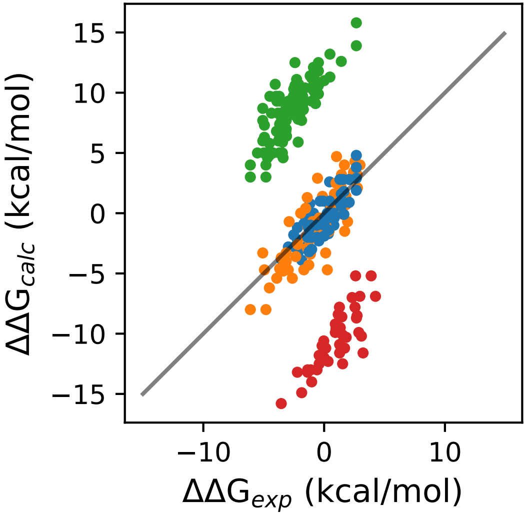

15
10
5
0
−5
−10
−15
−10
0
10
ΔΔG$_{calc}$ (kcal/mol)
ΔΔG$_{exp}$ (kcal/mol)
