# Supplementary material for: Training a force field for proteins and small molecules from scratch
Source: Chem Sci. 2026 Sep 17;17(42):20573–94. doi: 10.1039/d6sc02874h (PMC13585093; doi:10.1039/d6sc02874h)
Supplement: Supplementary file 2 [file SC-017-D6SC02874H-s001.zip › SI Figures/density_DHvap.pdf]

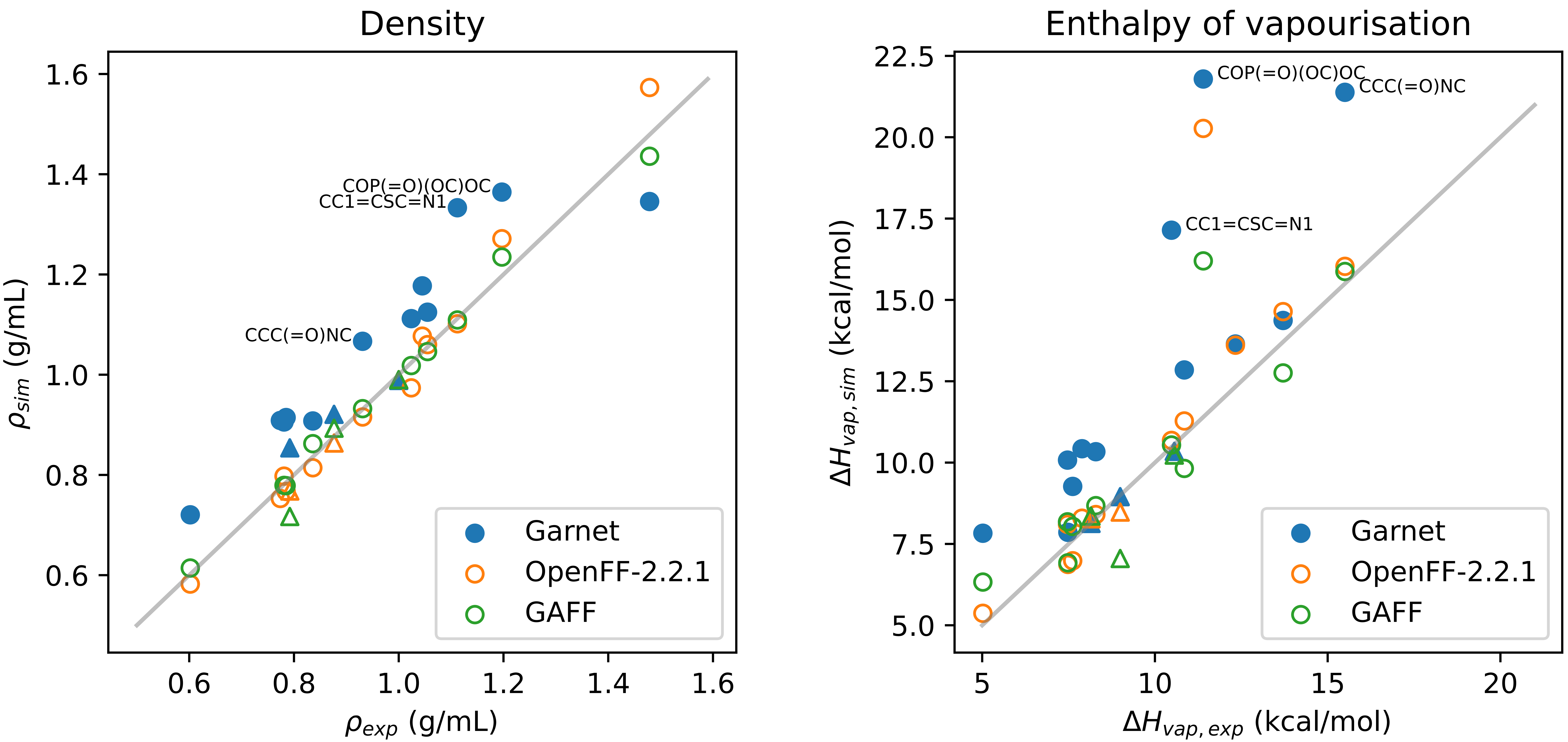

Density
COP(=O)(OC)OC
CC1=CSC=N1
CCC(=O)NC
ρ_{sim} (g/mL)
ρ_{exp} (g/mL)
Garnet
OpenFF-2.2.1
GAFF
Enthalpy of vapourisation
COP(=O)(OC)OC
CCC(=O)NC
CC1=CSC=N1
$\Delta H_{vap,sim}$ (kcal/mol)
$\Delta H_{vap,exp}$ (kcal/mol)
Garnet
OpenFF-2.2.1
GAFF
