# Supplementary material for: Training a force field for proteins and small molecules from scratch
Source: Chem Sci. 2026 Sep 17;17(42):20573–94. doi: 10.1039/d6sc02874h (PMC13585093; doi:10.1039/d6sc02874h)
Supplement: Supplementary file 2 [file SC-017-D6SC02874H-s001.zip › SI Figures/dipole_tensor_diffs.pdf]

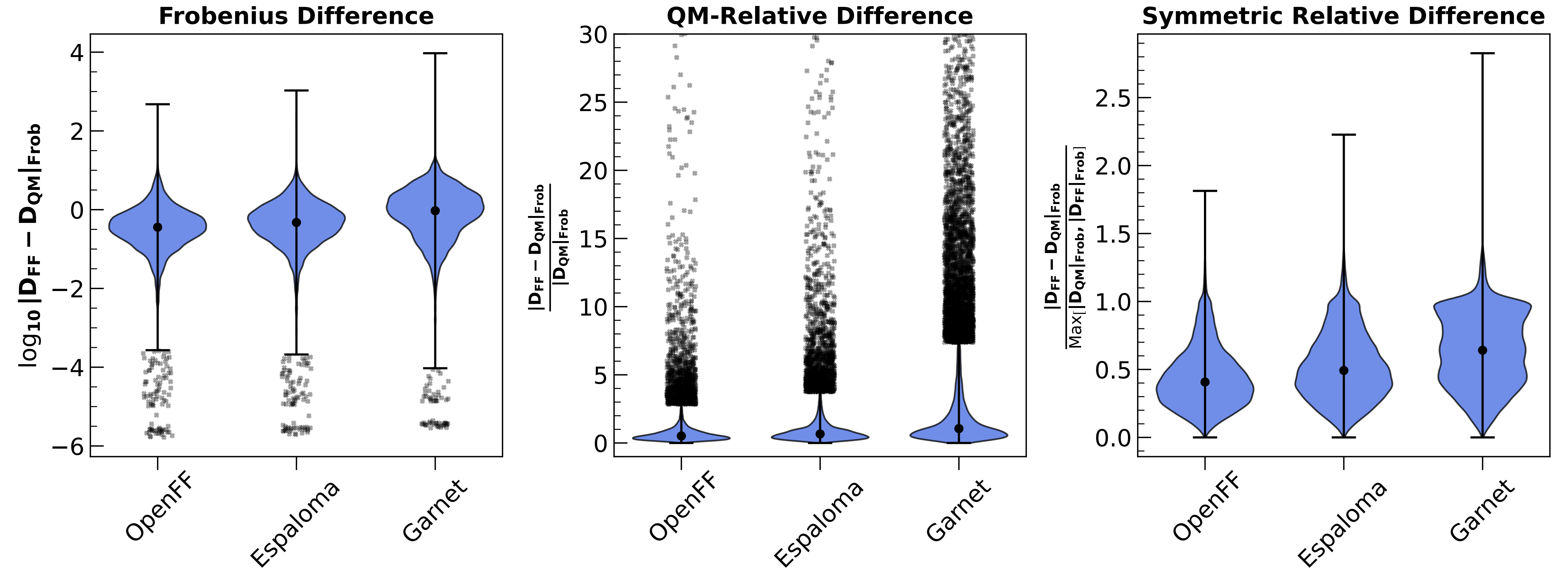

Frobenius Difference
QM-Relative Difference
Symmetric Relative Difference
$\log_{10}|\mathbf{D}_{FF} - \mathbf{D}_{QM}|_{Frob}$
$\frac{|\mathbf{D}_{FF} - \mathbf{D}_{QM}|_{Frob}}{|\mathbf{D}_{QM}|_{Frob}}$
$\frac{|\mathbf{D}_{FF} - \mathbf{D}_{QM}|_{Frob}}{\mathrm{Max}[|\mathbf{D}_{QM}|_{Frob}, |\mathbf{D}_{FF}|_{Frob}]}$
OpenFF
Espaloma
Garnet
