# Supplementary material for: Training a force field for proteins and small molecules from scratch
Source: Chem Sci. 2026 Sep 17;17(42):20573–94. doi: 10.1039/d6sc02874h (PMC13585093; doi:10.1039/d6sc02874h)
Supplement: Supplementary file 2 [file SC-017-D6SC02874H-s001.zip › SI Figures/embedding.pdf]

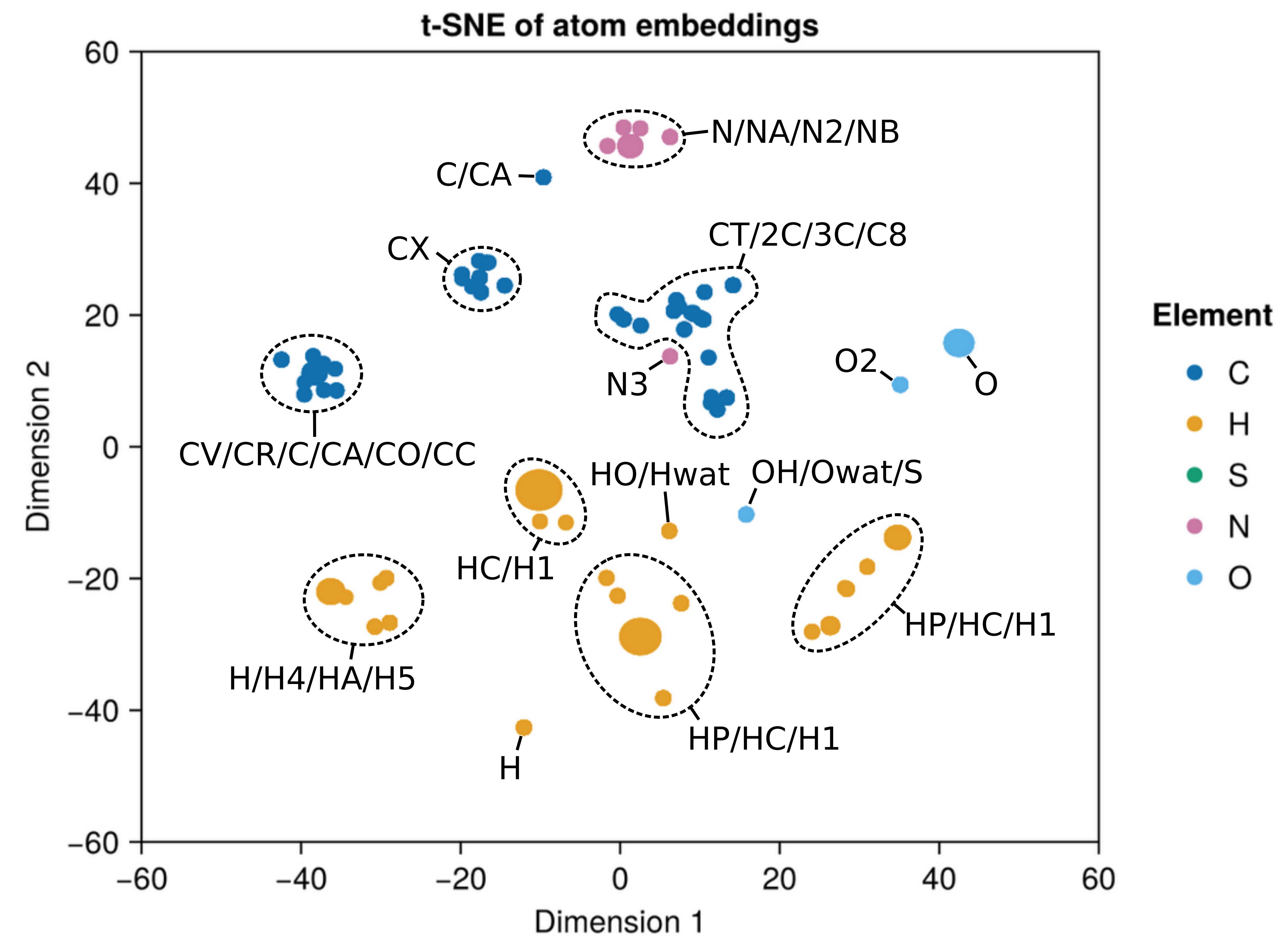

t-SNE of atom embeddings
N/NA/N2/NB
C/CA
CT/2C/3C/C8
CX
N3
O2
O
CV/CR/C/CA/CO/CC
HO/Hwat
OH/Owat/S
HC/H1
HP/HC/H1
H/H4/HA/H5
HP/HC/H1
H
Dimension 1
Dimension 2
60
40
20
0
−20
−40
−60
−60
−40
−20
0
20
40
60
Element
C
H
S
N
O
