# Supplementary material for: Training a force field for proteins and small molecules from scratch
Source: Chem Sci. 2026 Sep 17;17(42):20573–94. doi: 10.1039/d6sc02874h (PMC13585093; doi:10.1039/d6sc02874h)
Supplement: Supplementary file 2 [file SC-017-D6SC02874H-s001.zip › SI Figures/idp_density.pdf]

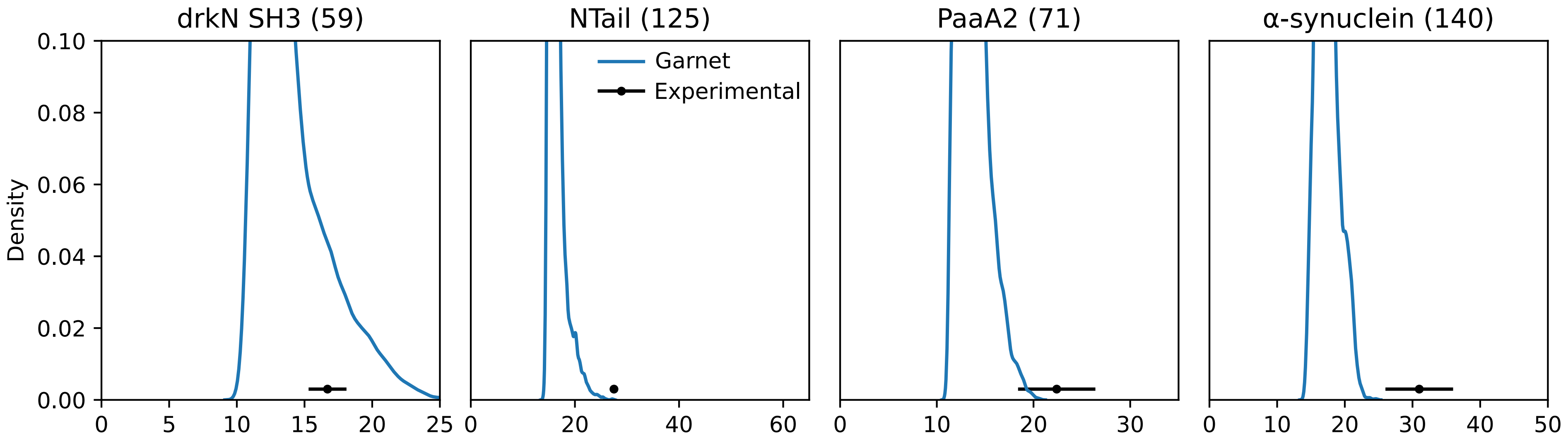

drkN SH3 (59)
NTail (125)
PaaA2 (71)
α-synuclein (140)
Density
Garnet
Experimental
0.00
0.02
0.04
0.06
0.08
0.10
0
5
10
15
20
25
0
20
40
60
0
10
20
30
0
10
20
30
40
50
