# Supplementary material for: Training a force field for proteins and small molecules from scratch
Source: Chem Sci. 2026 Sep 17;17(42):20573–94. doi: 10.1039/d6sc02874h (PMC13585093; doi:10.1039/d6sc02874h)
Supplement: Supplementary file 2 [file SC-017-D6SC02874H-s001.zip › SI Figures/parameters.pdf]

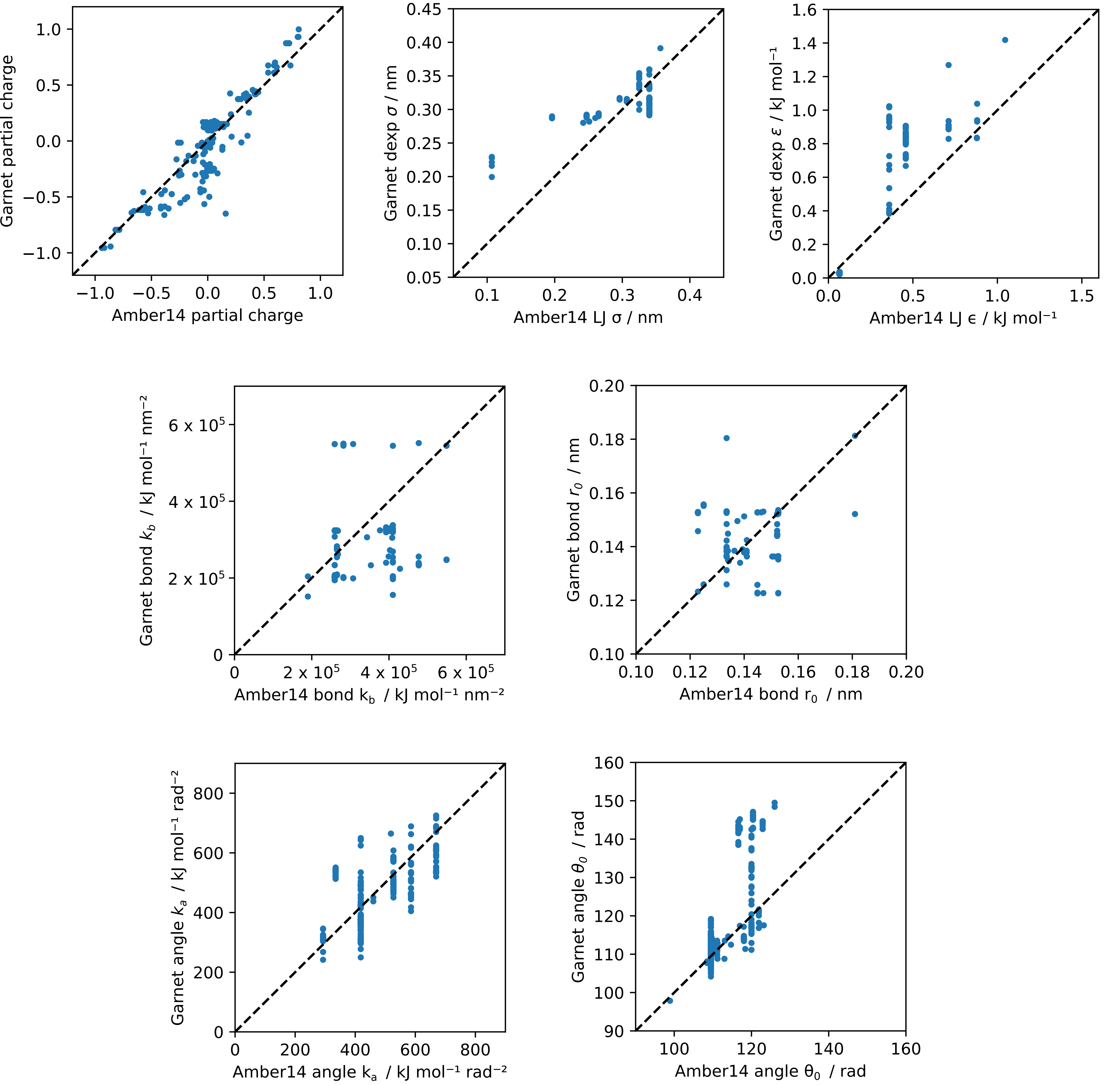
Garnet partial charge
Amber14 partial charge
Garnet dexp σ / nm
Amber14 LJ σ / nm
Garnet dexp ε / kJ mol⁻¹
Amber14 LJ ϵ / kJ mol⁻¹
Garnet bond k_b / kJ mol⁻¹ nm⁻²
Amber14 bond k_b / kJ mol⁻¹ nm⁻²
Garnet bond r_0 / nm
Amber14 bond r_0 / nm
Garnet angle k_a / kJ mol⁻¹ rad⁻²
Amber14 angle k_a / kJ mol⁻¹ rad⁻²
Garnet angle θ_0 / rad
Amber14 angle θ_0 / rad
