# Supplementary material for: Training a force field for proteins and small molecules from scratch
Source: Chem Sci. 2026 Sep 17;17(42):20573–94. doi: 10.1039/d6sc02874h (PMC13585093; doi:10.1039/d6sc02874h)
Supplement: Supplementary file 2 [file SC-017-D6SC02874H-s001.zip › SI Figures/rbfe_scatter_plots.pdf]

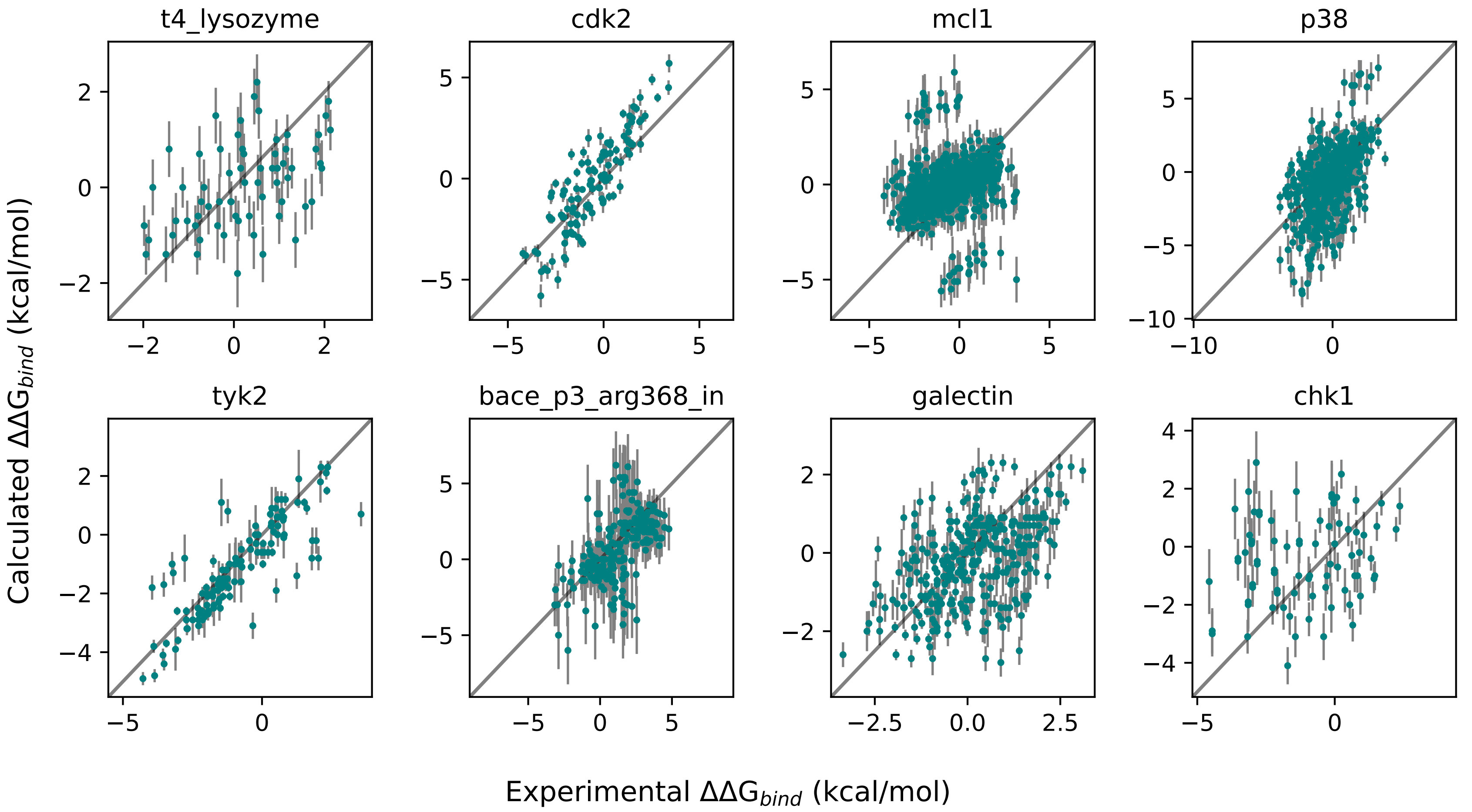

t4_lysozyme
cdk2
mcl1
p38
tyk2
bace_p3_arg368_in
galectin
chk1
Calculated ΔΔG$_{bind}$ (kcal/mol)
Experimental ΔΔG$_{bind}$ (kcal/mol)
