# Supplementary material for: Training a force field for proteins and small molecules from scratch
Source: Chem Sci. 2026 Sep 17;17(42):20573–94. doi: 10.1039/d6sc02874h (PMC13585093; doi:10.1039/d6sc02874h)
Supplement: Supplementary file 2 [file SC-017-D6SC02874H-s001.zip › SI Figures/rmsd.pdf]

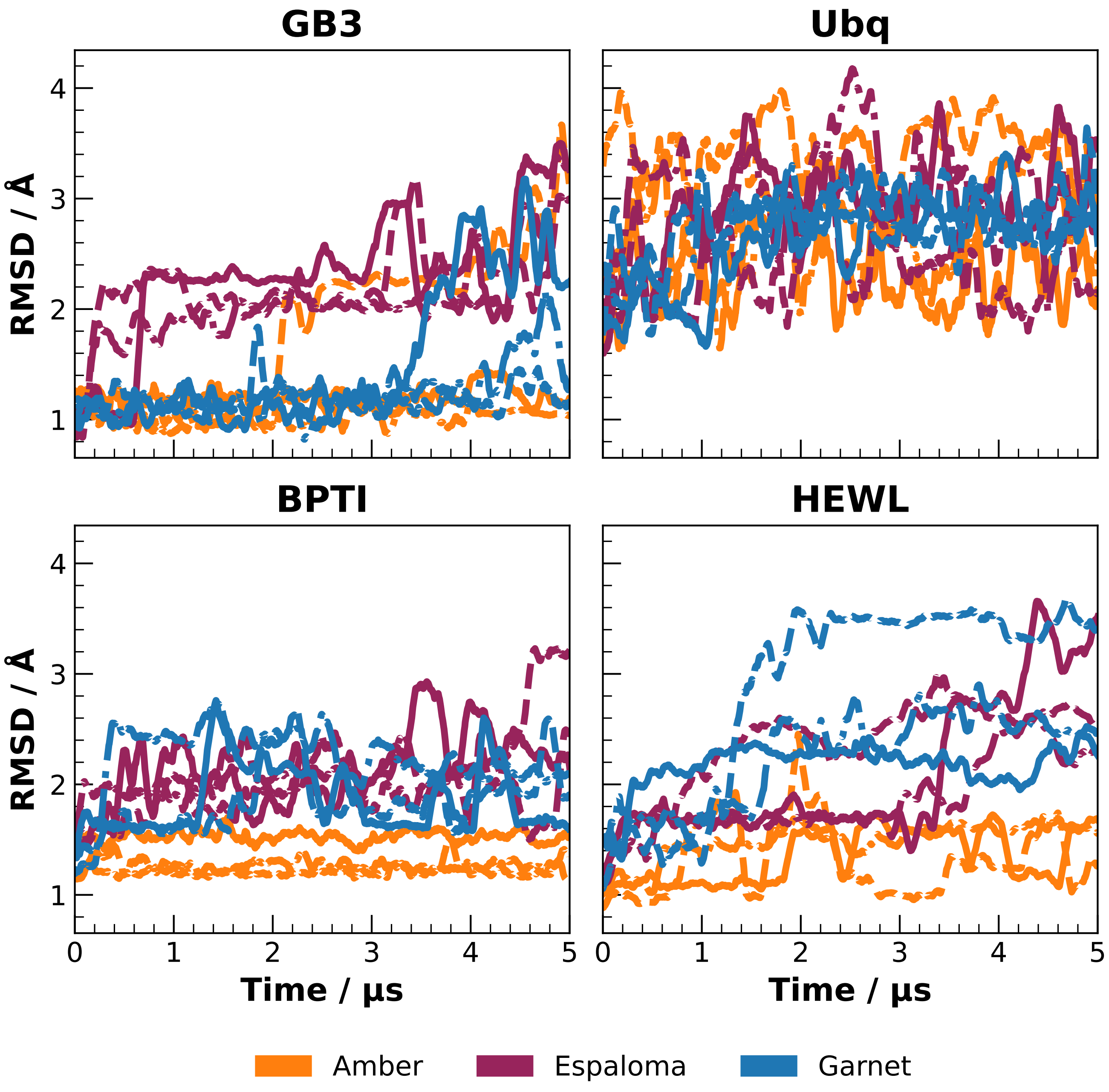
GB3
Ubq
BPTI
HEWL
RMSD / Å
RMSD / Å
Time / μs
Time / μs
1
2
3
4
0
1
2
3
4
5
Amber
Espaloma
Garnet
