# Supplementary material for: Training a force field for proteins and small molecules from scratch
Source: Chem Sci. 2026 Sep 17;17(42):20573–94. doi: 10.1039/d6sc02874h (PMC13585093; doi:10.1039/d6sc02874h)
Supplement: Supplementary file 2 [file SC-017-D6SC02874H-s001.zip › SI Figures/training.pdf]

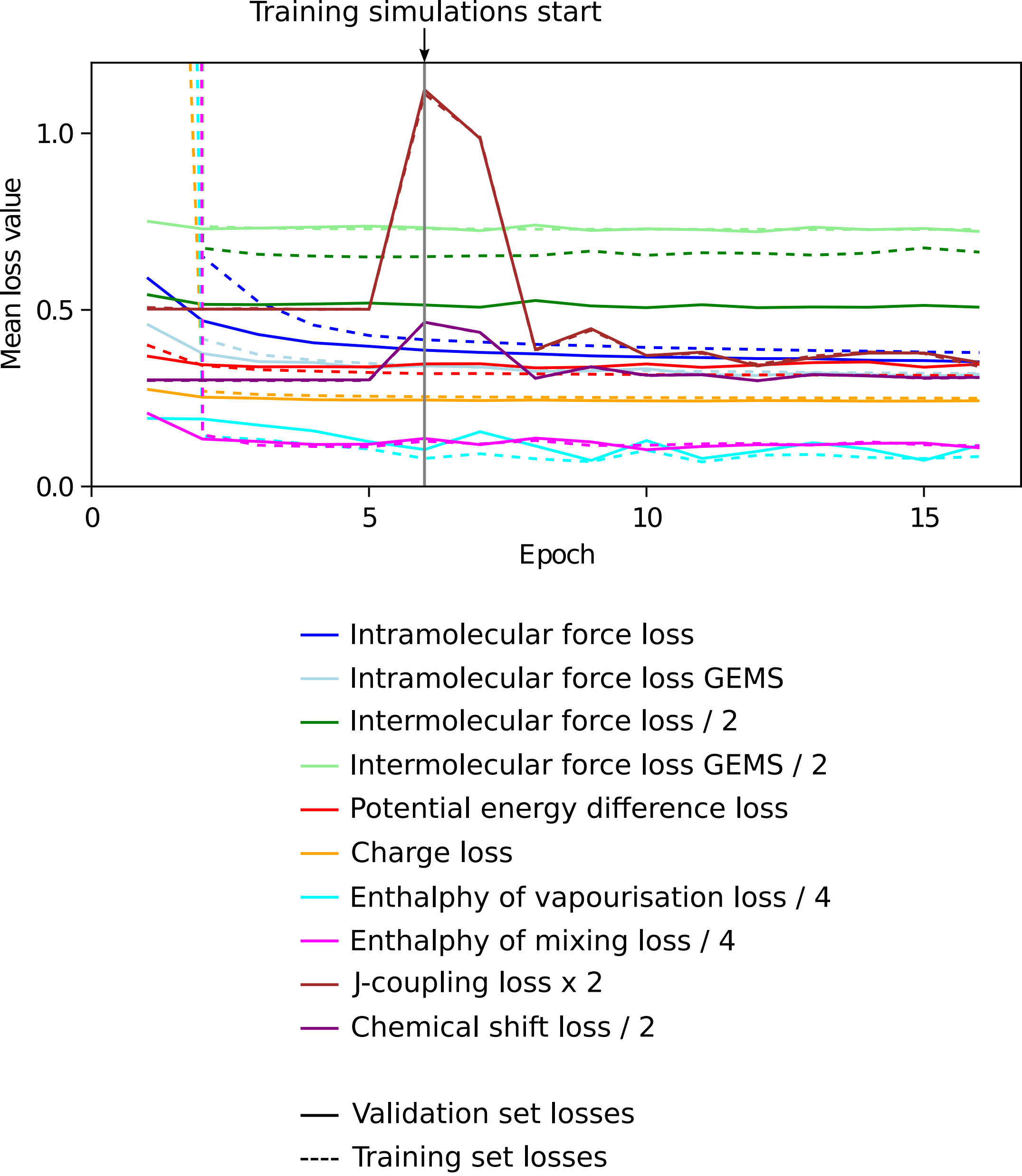
Training simulations start
Mean loss value
1.0
0.5
0.0
0
5
10
15
Epoch
Intramolecular force loss
Intramolecular force loss GEMS
Intermolecular force loss / 2
Intermolecular force loss GEMS / 2
Potential energy difference loss
Charge loss
Enthalphy of vapourisation loss / 4
Enthalphy of mixing loss / 4
J-coupling loss x 2
Chemical shift loss / 2
Validation set losses
Training set losses
